# A State-of-the-Art Review on Integral Transform Technique in Laser–Material Interaction: Fourier and Non-Fourier Heat Equations

**DOI:** 10.3390/ma14164733

**Published:** 2021-08-22

**Authors:** Mihai Oane, Muhammad Arif Mahmood, Andrei C. Popescu

**Affiliations:** 1 National Institute for Laser, Plasma and Radiation Physics (INFLPR), Magurele, 077125 Ilfov, Romania; mihai.oane@inflpr.ro; 2 Faculty of Physics, University of Bucharest, Magurele, 077125 Ilfov, Romania

**Keywords:** Fourier heat equation, non-Fourier heat equation, integral transform technique, generalized solutions, heat equation components, MATHEMATICA software user-defined codes

## Abstract

Heat equations can estimate the thermal distribution and phase transformation in real-time based on the operating conditions and material properties. Such wonderful features have enabled heat equations in various fields, including laser and electron beam processing. The integral transform technique (ITT) is a powerful general-purpose semi-analytical/numerical method that transforms partial differential equations into a coupled system of ordinary differential equations. Under this category, Fourier and non-Fourier heat equations can be implemented on both equilibrium and non-equilibrium thermo-dynamical processes, including a wide range of processes such as the Two-Temperature Model, ultra-fast laser irradiation, and biological processes. This review article focuses on heat equation models, including Fourier and non-Fourier heat equations. A comparison between Fourier and non-Fourier heat equations and their generalized solutions have been discussed. Various components of heat equations and their implementation in multiple processes have been illustrated. Besides, literature has been collected based on ITT implementation in various materials. Furthermore, a future outlook has been provided for Fourier and non-Fourier heat equations. It was found that the Fourier heat equation is simple to use but involves infinite speed heat propagation in comparison to the non-Fourier heat equation and can be linked with the Two-Temperature Model in a natural way. On the other hand, the non-Fourier heat equation is complex and involves various unknowns compared to the Fourier heat equation. Fourier and Non-Fourier heat equations have proved their reliability in the case of laser–metallic materials, electron beam–biological and –inorganic materials, laser–semiconducting materials, and laser–graphene material interactions. It has been identified that the material properties, electron–phonon relaxation time, and Eigen Values play an essential role in defining the precise results of Fourier and non-Fourier heat equations. In the case of laser–graphene interaction, a restriction has been identified from ITT. When computations are carried out for attosecond pulse durations, the laser wavelength approaches the nucleus-first electron separation distance, resulting in meaningless results.

## 1. Introduction

Various engineering problems can be modeled using partial differential equations (PDEs) with initial and boundary conditions. For this purpose, numerical approaches, including finite element, finite difference, boundary element, and spectral techniques, are usually applied [1]. The integral transform technique (ITT) is mainly applied to solve linear system problems [2]. A generalized ITT was recently used to resolve numerous linear and non-linear models [3]. All methods reduce the PDEs to a set of ordinary differential equations (ODEs) that can be solved via well-known techniques. For finite difference and element techniques, the field variables are considered in a limited number of points; however, in the boundary elements approach, the number of boundary elements points is minimal. Furthermore, the spectral technique yields a solution in terms of short series numbers. Besides, ITT utilizes “Eigen Function” expansion.

The first law of thermodynamics, also known as the law of conservation of energy, explains that the total energy of an isolated system is constant; energy can be transformed from one form to another but can be neither created nor destroyed [4]. The second law of thermodynamics is about the “quality” of energy. It states that as energy is transferred or transformed, more and more of it is wasted. The second law also states that any isolated system has a natural tendency to degenerate into a more disordered state [4]. It is worth mentioning that all valid heat equations, including Fourier and non-Fourier, obey the first and second laws of thermodynamics.

In this study, the authors have presented the integral transform technique on Fourier and non-Fourier heat equations. We presented the generalized solutions of both techniques in laser–material interaction for the experimentalists to perform simulations before carrying out the actual experiments. We also discussed the various sections of Fourier and non-Fourier heat equations. To further facilitate the experimentalists in this field, we developed friendly user-defined software in MATHEMATICA and provided it within the manuscript for the audience. To the best of the authors’ knowledge, this is the first study that compiles ITT implementation on Fourier and non-Fourier heat equations along with the user-defined codes in the field of experimental physics. 

In this review paper, ITT is illustrated as a reliable tool for solving heat equations engineering problems. This technique converts non-linear PDEs to a coupled non-linear ODEs so that they can be solved numerically. ITT on heat equations has been classified into (a) Fourier and (b) non-Fourier heat equations. The generalized solutions of Fourier and non-Fourier heat equations have been derived and discussed. The components of heat equations have been described in detail. Various applications of ITT have been illustrated. Besides, user-defined codes have been provided with explanations in the case of “MATHEMATICA” software programs. After reading this article, one will be able to implement ITT, for Fourier and non-Fourier, in various engineering applications.

### 1.1. Generalities in Heat Equation: Fourier Heat Equation Formalism and Its Solution

Consider a body at temperature, *T*(*x*,*y*,*z*), at the position *M*(*x*,*y*,*z*), and time (*t*). The temperature rises and falls in various body areas, moving from higher to lower temperatures. A small area (Δ*S*) around *M*(*x*,*y*,*z*) can be considered. The section to be heated (Δ*Q*) is proportional to (Δ*t*Δ*S*) and normal-derivative $\left(\frac{\partial T}{\partial n}\right)$, as:

(1) $$\Delta Q=-k\left(x,y,z\right)\Delta t\Delta S\frac{\partial T}{\partial n}$$

Here, *k*(*x*,*y*,*z*) and *n* are the thermal conductivity and vector perpendicular to the Δ*S* and temperature declination orientation, respectively. In terms of thermal conductivity, it has been assumed that the solid-body probe is functioning isotopically. Here, heat passing through a surface area per unit time is denoted as “*q*.” Now, Equation (1) can be expressed as:

(2) $$q=-k\frac{\partial T}{\partial n}.$$

To solve the heat equation, consider a random volume (*V*) covered by a surface (*S*) and perceive the temperature change within the interval time interval (*t*_1_–*t*_2_). After taking into account the above-defined explanations, one will obtain the following expression:

(3) $$Q_{1}=-\int_{t_{2}}^{t_{1}}dt\int_{S}^{\;}\int \;k\left(x,y,z\right)\frac{\partial T}{\partial n}dS.$$

For a given element volume (Δ*V*), the heat within the element is given as:

(4) $$\Delta Q_{2}=\left[T\left(x,y,z,t+\Delta t\right)-T\left(x,y,z,t\right)\right]c\left(x,y,z\right)\rho \left(x,y,z\right)\Delta V,$$

where *c*(*x*,*y*,*z*) and ρ(x,y,z) are specific heat and density, respectively, for an element volume, it is essential to change from *t*_1_ to *t*_2_, expressed as:

(5) $$\Delta T=T\left(x,y,z,t_{2}\right)-T\left(x,y,z,t_{1}\right)$$

Therefore

(6) $$Q_{2}={∭}_{V}\left[T\left(x,y,z,t_{2}\right)-T\left(x,y,z,t_{1}\right)\right]c\rho dV.$$

Consequently:

(7) $$T\left(x,y,z,t_{2}\right)-T\left(x,y,z,t_{1}\right)=\int_{t_{1}}^{t_{2}}\frac{\partial T}{\partial t}dt.$$

Now, let us assume that there is a heat source within the solid body. In a unit volume per time unit, the source causes the heat absorbance or release, denoted by *A*(*x*,*y*,*z*,*t*). The heat absorbance or release resulted from the volume (*V*) during the time duration $\left(t_{1},t_{2}\right)$ is given as:

(8) $$Q_{3}=\int_{t_{1}}^{t_{2}}dt{∭}_{V}A\left(x,y,z,t\right)dV.$$

Noticeably, thermal equilibrium is assumed within the volume *V*, as:

(9) $$Q_{2}=Q_{1}+Q_{3}.\;$$

It infers:

(10) $$\int_{t_{2}}^{t_{1}}dt\int^{\;}\int_{V}\int^{\;}c\rho \frac{\partial T}{\partial t}dV=-\int_{t_{2}}^{t_{1}}dt\iint_{S}k\frac{\partial T}{\partial n}dS+\int_{t_{2}}^{t_{1}}dt{∭}_{V}A\left(x,y,z,t\right)dV.$$

By utilizing the Gauss–Ostrogradsky formalism, one must achieve:

(11) $$\int_{t_{2}}^{t_{1}}dt\int^{\;}\int_{V}\int^{\;}[c\rho \frac{\partial T}{\partial t}dV-div(k\cdot grad\;T)-A\left(x,y,z,t\right)]dV=0.$$

Equation (11) describes the thermal-field process that evolved when radiations interact with a solid. Exact analytical results can be achieved if a series of generalizations concerning temporal and spatial distributions for a given radiations source and the geometry are made. If the configurations of the radiation source and the geometry to be irradiated become more severe, it is very tough to determine an analytical solution and the thermal distribution, T(x,y,z,t), expression can be evaluated numerically. A three-dimensional (3D) heat equation is generally expressed as:

(12) $$\rho C\frac{\partial T}{\partial t}=\frac{\partial }{\partial x}\left(k\frac{\partial T}{\partial x}\right)+\frac{\partial }{\partial y}\left(k\frac{\partial T}{\partial y}\right)+\frac{\partial }{\partial z}\left(k\frac{\partial T}{\partial z}\right)+A\left(x,y,z,t\right).$$

Here, *x*, *y*, and *z* are the universal Cartesian coordinates, *T* is the temperature, *t* is the time, *k* is the thermal conductivity, *C* is the material’s specific heat, *ρ* is the material’s density, and *A*(*x*,*y*,*z*,*t*) is the heat source. Once the solid body is assumed to be consistent and isotropous, Equation (12) reduces to:

(13) $$\nabla^{2}T-\frac{1}{\gamma }\frac{\partial T}{\partial t}=-\frac{A\left(x,y,z,t\right)}{k}$$

where γ (=k/ρC) is the thermal diffusivity. For the steady-state condition ($\frac{\partial T}{\partial t}=0$), Equation (13) reduces to:

(14) $$\nabla^{2}T=-\frac{A\left(x,y,z\right)}{k},\;$$

If no external heating source is applied, Equation (13) becomes:

(15) $$\nabla^{2}T=\frac{1}{\gamma }\frac{\partial T}{\partial t}.$$

In the case of stationary and no heating source, one achieves the following term:

(16) $$\nabla^{2}T=0$$

Equation (10) has an infinite heat-wave speed, and the formalism is known as the “Fourier heat” equation.

(17) $$\nabla^{2}T=0$$

In the current study, a cylinder has been considered. It is the reason why the authors used cylindrical coordinates. Equation (12) in terms of cylindrical coordinates (*r*,*z*,*ϕ*) can be written as:

(18) $$\frac{\partial^{2}T\left(r,z,\phi ,t\right)}{\partial r^{2}}+\frac{1}{r}\frac{\partial T\left(r,z,\phi ,t\right)}{\partial r}+\frac{\partial^{2}T\left(r,z,\phi ,t\right)}{\partial z^{2}}+\frac{1}{r^{2}}\frac{\partial^{2}T\left(r,z,\phi ,t\right)}{\partial \phi^{2}}-\frac{1}{\gamma }\frac{\partial T\left(r,z,\phi ,t\right)}{\partial t}=-\frac{A\left(r,z,\phi ,t\right)}{k}.$$

The boundary conditions are:

(19) $$K{\frac{\partial T\left(r,z,\phi ,t\right)}{\partial r}|}_{r=b}+hT\left(b,z,\phi ,t\right)=0.$$

(20) $$K{\frac{\partial T\left(r,z,\phi ,t\right)}{\partial r}|}_{z=0}+hT\left(r,0,\phi ,t\right)=0.$$

(21) $$K{\frac{\partial T\left(r,z,\phi ,t\right)}{\partial r}|}_{z=a}+hT\left(r,a,\phi ,t\right)=0.$$

Furthermore, the periodic boundary condition is:

(22) T(r,0,z,t)=T(r,2π,z,t).

After supposing that laser beam is emitting a beam in the transversal electromagnetic mode (TEM_mn_), the thermal distribution variation *T(r, z, φ, t*) can be determined as [5]:

(23) $$T\left(r,\phi ,z,t\right)=\sum_{m,n}\sum_{i=1}^{\infty }\sum_{l=0}^{\infty }\sum_{j=1}^{\infty }{\hat{f}}_{2l}\left(\mu_{il},\lambda_{j},l\right)g\left(\mu_{il},\lambda_{j},t\right)K_{r}\left(\mu_{il},r\right)K_{\phi }\left(2l,\phi \right)K_{z}\left(\lambda_{j},z\right)\;\;\;\;\;\;\;\;\;\;\;\;+\sum_{m,n}\sum_{i=1}^{\infty }\sum_{l=0}^{\infty }\sum_{j=1}^{\infty }{\hat{f}}_{2l-1}\left(\mu_{il},\lambda_{j},l\right)g\left(\mu_{il},\lambda_{j},t\right)K_{r}\left(\mu_{il},r\right)K_{\phi }\left(2l-1,\phi \right)K_{z}\left(\lambda_{j},z\right)$$

Equation (23) is the solution based on the boundary conditions given in Equations (19)–(22). Furthermore:

(24) $${\overset{\wedge }{f}}_{2l}\left(\mu_{il},\lambda_{j},l\right)=\frac{1}{k\pi C_{il}C_{j}}\int_{0}^{a}\alpha_{s}e^{-\alpha_{s}z}K_{z}\left(\lambda_{j},z\right)dz\int_{0}^{b}\int_{0}^{2\pi }I_{mn}\left(r,\phi \right)rK_{r}\left(\mu_{il},r\right)K_{\phi }\left(2l,\phi \right)drd\phi .$$

(25) $${\overset{\wedge }{f}}_{2l-1}\left(\mu_{il},\lambda_{j},l\right)=\frac{1}{k\pi C_{il}C_{j}}\int_{0}^{a}\alpha_{s}e^{-\alpha_{s}z}K_{z}\left(\lambda_{j},z\right)dz\int_{0}^{b}\int_{0}^{2\pi }I_{mn}\left(r,\phi \right)rK_{r}\left(\mu_{il},r\right)K_{\phi }\left(2l-1,\phi \right)drd\phi .$$

Here, ${\overset{\wedge }{f}}_{2l}\left(\mu_{il},\lambda_{j},l\right)$ and ${\overset{\wedge }{f}}_{2l-1}\left(\mu_{il},\lambda_{j},l\right)$ are the source terms of the solution. The term “A(r,z,ϕ,t)” is the laser beam interaction with the material defined in correspondence with the Lambert–Beer law. It can be identified in the final solution of the heat equation given by ${\overset{\wedge }{f}}_{2l}\left(\mu_{il},\lambda_{j},l\right)$ and ${\overset{\wedge }{f}}_{2l-1}\left(\mu_{il},\lambda_{j},l\right)$ source terms of the solution. We also have:

(26) $$I_{mn}\left(x,y\right)=I_{0mn}{\left\{H_{m}\left(\frac{\sqrt{2x}}{w}\right)H_{n}\left(\frac{\sqrt{2x}}{w}\right)exp\left[-\left(\frac{x^{2}+y^{2}}{w^{2}}\right)\right]\right\}}^{2}.$$

(27) $$g\left(\mu_{il},\lambda_{j},t\right)=\frac{1}{\left(\mu_{il}^{2}+\lambda_{j}^{2}\right)\left[1-e^{-\beta_{ijl}^{2}t}-\left(1-e^{-\beta_{ijl}^{2}\left(t-t_{0}\right)}\right)h\left(t-t_{0}\right)\right]}$$

(28) $$\beta_{ilj}^{2}=\gamma \left(\mu_{il}^{2}+\lambda_{j}^{2}\right).$$

Here, $g\left(\mu_{il},\lambda_{j},t\right)$ and $\beta_{ilj}^{2}$ are the part of the temporal part of the solution given by direct and inverse Laplace transform. Furthermore, *h*(*t* − *t*_0_), *t*_0*,*_ and *a_s_* are the Heaviside function, laser–material interaction time, and linear absorption coefficient. Besides, *K_r_*(*m_il_,r*)*, K_φ_*(2*l,φ*)*, K_φ_*(2*l*−1*,φ*) and *K_z_*(*λ_j_,z*) can be recognized as the “Eigen Functions” for the Eigen Values *m_il_*, 2*l*, 2*l−*1 and *λ_j_*. Therefore:

*K_r_*(*m_il_*,*r*) = *J*(*m_il_r*). (29)

*K_φ_*(2*l*,*φ*) = *cos*(*lφ*). (30)

*K_φ_*(2*l*−1*,φ*) = sin(*lφ*). (31)

*K_z_*(*λ_j_*,*z*) = *cos*(*λ_j_z*) + (*h*/*kλ_j_*)*sin*(*λ_j_z*). (32)

Besides, *J* is the zero^th^-order Bessel function, and *C_il_* and *C_j_* are the normalizing coefficients. It is worthy of mentioning that Eigen Values are dependent on the boundary conditions.

### 1.2. Non-Fourier Heat Equation Formalism and Its Solution

Consider the general form of the non-Fourier transform heat equation for laser–solid interaction. Keeping the standardized notation, the heat source term is expressed as:

(33) $$A\left(x,y,z,t\right)=\{\begin{array}{l} \alpha I_{0}e^{-\alpha x};\;0\leq t\leq \tau \\ 0;\;t<0\;\;and\;t>\tau \end{array}$$

Here, the Cartesian coordinates have been considered as a sample in parallelepiped shape is irradiated with a laser beam. The following equation shows the non-Fourier heat equation:

(34) $$\frac{\partial^{2}T}{\partial x^{2}}+\frac{\partial^{2}T}{\partial y^{2}}+\frac{\partial^{2}T}{\partial z^{2}}-\frac{1}{\gamma }\frac{\partial T}{\partial t}-\frac{\tau_{0}}{\gamma }\frac{\partial^{2}T}{\partial t^{2}}=-\frac{A\left(x,y,z,t\right)}{K}\left(1+\tau_{0}\delta \left(t\right)\right).$$

Here, γ is the thermal diffusivity, and *K* is the thermal conductivity. After implementing ITT, one will obtain the following expression [5]:

(35) $$\mu_{i}^{2}\hat{T}+\beta_{l}^{2}\hat{T}+\lambda_{j}^{2}\hat{T}+\frac{1}{\gamma }\frac{\partial \hat{T}}{\partial t}+\frac{\tau_{0}}{\gamma }\frac{\partial^{2}\hat{T}}{\partial t^{2}}=\frac{\hat{A}\left(x,\;\;y,\;\;z,\;t\right)}{K}\left(1+\tau_{0}\delta \left(t\right)\right).$$

ITT is interpreted as a powerful general-purpose semi-analytical/numerical method. The method transforms partial differential equation models to a coupled system of ordinary differential equations. The implementation of this technique can be identified in-detail from Refs. [6,7]. Here, one should note that *a*, *b* and *c* are the length, width, and height of the substrate, with:

(36) $$f\left(\mu_{i},\;\beta_{l},\lambda_{j},\;t\right)=\int_{0}^{a}\int_{0}^{b}\int_{0}^{c}{\hat{K}}_{x}\left(\mu_{i},x\right){\hat{K}}_{y}\left(\beta_{l},y\right){\hat{K}}_{Z\;}\left(\lambda_{j},z\right)\frac{\hat{A}\left(x,\;\;y,\;\;z,\;\;t\right)}{K}\left(1+\tau_{0}\delta \left(t\right)\right).$$

Here, $f\left(\mu_{i},\;\beta_{l},\lambda_{j},\;t\right)$ and ${\hat{K}}_{x}\left(\mu_{i},\;x\right)$ are the source terms of a solution, ${\hat{K}}_{x}\left(\mu_{i},x\right),\;{\hat{K}}_{y}\left(\beta_{l},y\right)$ and ${\hat{K}}_{Z\;}\left(\lambda_{j},z\right)$ are the “Eigen Functions.” The first “Eigen Function (${\hat{K}}_{x}\left(\mu_{i},x\right),$) is calculated as:

(37) $${\hat{K}}_{x}\left(\mu_{i},\;x\right)=\frac{1}{c_{i}}K_{x}\left(\mu_{i},x\right).$$

Furthermore, in the case of the ITT, we have the following expression [5]:

(38) $$\frac{\partial^{2}{\hat{K}}_{x}}{\partial x^{2}}+\lambda {\hat{K}}_{x}=0.$$

The boundary conditions along with the *x*-axis are given as:

(39) $${\left[k\left(\frac{\partial {\hat{K}}_{x}}{\partial x}\right)-h{\hat{K}}_{x}\right]}_{x=0}=0.$$

(40) $${\left[k\left(\frac{\partial {\hat{K}}_{x}}{\partial x}\right)+h{\hat{K}}_{x}\right]}_{x=a}=0.$$

The “Eigen Function” can be defined as [5]:

(41) $${\hat{K}}_{x}\left(\mu_{i},x\right)=\frac{1}{C_{i}}\left(\cos\left(\mu_{i}x\right)+\frac{h}{k\mu_{i}}\sin\left(\mu_{i}x\right)\right).$$

where $C_{i}$ is the normalizing constant, calculated as [5]:

(42) $$C_{i}=\int_{0}^{a}{\hat{K}}_{x}^{2}\left(\mu_{i},x\right)dx.$$

The expression for “Eigen Values”$\left(\mu_{i}\right)$ is given as [5]:

(43) $$2\cot\left(\mu_{i}a\right)=\frac{\mu_{i}k}{h}-\frac{h}{k\mu_{i}}.$$

Note that:

(44) $$\eta_{ilj}^{2}=\frac{1}{\gamma^{2}}-4\left(\mu_{i}^{2}+\beta_{l}^{2\;}+\lambda_{j}^{2}\right)\frac{\tau_{0}}{\gamma }.$$

It is worthy to mention that the other two “Eigen Values” have also been calculated in the same manner. The temporal part of the generalized solution is obtained via direct and inverse Laplace transform and is expressed as [5]:

(45) $$\begin{array}{ll} g\left(\mu_{i},\beta_{l},\lambda_{j},t\right)= & e^{\left(\frac{-\;1\;-\;\gamma \;\eta_{ilj}}{2\tau_{0}}\right)t}C_{1}+e^{\left(\frac{-\;1+\;\gamma \eta_{ilj}}{2\tau_{0}}\right)t}C_{2}\\ & -(4\frac{\tau_{0}A}{\gamma \;K}(\eta_{ilj}-\mu_{i}^{2}e^{\left(\frac{-\;1\;-\;\gamma \eta_{ilj}}{2\tau_{0}}\right)t}\tau_{0}Unit\;Step\;\left[t\right]-\;\beta_{l}^{2}e^{\left(\frac{-\;1\;-\gamma \eta_{ilj}}{2\tau_{0}}\right)t}\tau_{0}Unit\;Step\;\left[t\right]\\ & -\;\lambda_{j}^{2}e^{\left(\frac{-\;1-\;\gamma \eta_{ilj}}{2\tau_{0}}\right)t}\tau_{0}Unit\;Step\;\left[t\right]+\;\mu_{i}^{2}e^{\left(\frac{-\;1+\;\gamma \eta_{ilj}}{2\tau_{0}}\right)t}\tau_{0}Unit\;Step\;\left[t\right]\\ & +\;\beta_{l}^{2}e^{\left(\frac{-\;1+\;\gamma \eta_{ilj}}{2\tau_{0}}\right)t}\tau_{0}Unit\;Step\;\left[t\right]\\ & +\;\lambda_{j}^{2}e^{\left(\frac{-\;1+\;\gamma \eta_{ilj}}{2\tau_{0}}\right)t}\tau_{0}Unit\;Step\;\left[t\right]))\eta_{ilj}^{-1\;}{\left(\left(-\frac{1}{\gamma }+\;\eta_{ilj}\right)\left(\frac{1}{\gamma }+\;\eta_{ilj}\right)\right)}^{-1}. \end{array}$$

The unknown constants, $C_{1}$ and $C_{2}$, in the above solution, can be assessed using supplementary boundary conditions, as:

(46) $$T\left(x,y,z,0\right)=0\;\;\Rightarrow C_{1}+C_{2}=-\frac{4\tau_{0}A}{\gamma K\left(\eta_{ilj}^{2}-\frac{1}{\gamma^{2}}\right)}.$$

(47) $$T\left(\infty ,y,z,t\right)=0\;\Rightarrow C_{1}=-C_{2}e^{\frac{\;\gamma \eta_{ilj}t\;}{\tau_{0}}}.$$

One will obtain:

(48) $$C_{2}=\frac{-4\tau_{0}A}{\gamma K\left(1-e^{\frac{\;\gamma \eta_{ilj\;}t}{\tau_{0}}}\right)\left(\eta_{ilj}^{2}-\frac{1}{\gamma^{2}}\right)}$$

(49) $$C_{1}=\frac{4\tau_{0}Ae^{\frac{\gamma \eta_{ilj\;}t}{\tau_{0}}}}{\gamma K\left(1-\;e^{\frac{\;\gamma \eta_{ilj\;}t}{\tau_{0}}}\right)\left(\eta_{ilj}^{2}-\frac{1}{\gamma^{2}}\right)}$$

Note that the boundary conditions provided in the Fourier section (Equations (19)–(22)) also imply in the non-Fourier section taking into consideration Cartesian Coordinates. Besides, Equations (39) and (40) are the boundary conditions along the *x*-axis during irradiation. The same type of boundary conditions applies for y- and z-axes. Furthermore, Equations (46) and (47) are the supplementary boundary conditions. It is worth mentioning that T represents the temperature variation, not the absolute temperature. The current study focuses on ITT’s implementation in the non-Fourier heat equation, also known as the Cattaneo–Vernotte equation. The current study’s authors made the first attempt to link the ITT with the non-Fourier heat equation, as presented in Ref. [8].

Fourier and non-Fourier heat equations are valid for equilibrium and non-equilibrium thermodynamics. However, in non-equilibrium thermodynamics, one can implement these solutions for the femtosecond time domain [9]. Besides these techniques, there are various other frameworks, including extended irreversible thermodynamics, generic thermodynamics, internal variables, and rational extended thermodynamics. Jou et al. [10] presented a new formulation of non-equilibrium thermodynamics, known as extended irreversible thermodynamics, which has fueled increasing attention. Evolution equations for these fluxes were obtained starting from a hypothesis and using methods similar to classical irreversible thermodynamics. These equations were reduced to the classical constitutive laws in the limit of slow phenomena. Still, they may also be applied to fast phenomena, such as second sound in solids, ultrasound propagation, or generalized hydrodynamics. In contrast with the classical theory, extended thermodynamics lead to hyperbolic equations with finite propagation speeds for thermal and viscous signals. The results of the macroscopic theory were confirmed by the kinetic theory of gases and non-equilibrium statistical mechanics. The presented theory is particularly useful for studying the thermodynamics of non-equilibrium steady states and systems with long relaxation times, such as viscoelastic media or systems at low temperatures. There is no difficulty in formulating the theory in the relativistic context. Szucs et al. [11] simultaneously applied the methodology of non-equilibrium thermodynamics with internal variables (NET-IV) and the framework of general equation for the non-equilibrium reversible–irreversible coupling (GENERIC). They demonstrated that, in heat conduction theories, entropy current multipliers could be interpreted as relaxed state variables. Fourier’s law and its various extensions–the Maxwell–Cattaneo–Vernotte, Guyer–Krumhansl, Jeffreys type, Ginzburg–Landau (Allen–Cahn) type, and ballistic-diffusive–heat conduction equations were derived in both formulations. They proved that the results might pave the way for microscopic/multiscale understanding of beyond-Fourier heat conduction and open new ways for numerical simulations of heat-conduction problems. Kovacs et al. [12] discussed two different theories: the kinetic-theory-based rational extended thermodynamics (RET) and non-equilibrium thermodynamics with internal variables (NET-IV). It was shown how NET-IV structure is related to RET and how the compatibility between them can be acquired. In another study by Van and Kovacs [13], a comparison of thermodynamic and variational techniques was presented. They found that the second law alone can effectively construct evolution equations for both dissipative and non-dissipative processes.

Furthermore, Jou [14] considered a few conceptual questions on extended thermodynamics to contribute to a higher contact between rational extended thermodynamics and extended irreversible thermodynamics. Both theories take several fluxes as independent variables. Still, they differ in the formalism dealing with the exploitation of the second principle (rational thermodynamics in the first one and classical irreversible thermodynamics in the second one). Rational extended thermodynamics is more restricted in the range of systems to be analyzed, but it is able to obtain a wider number of restrictions and deeper specifications from the second law. By contrast, extended irreversible thermodynamics is more phenomenological, its mathematical formalism is more elementary, but it may deal with a wider diversity of systems, although with less detail. Further comparison and dialogue between both branches of extended thermodynamics would be useful for a fuller deployment and deepening. Besides these two approaches, one should also consider the Hamiltonian approach, formalisms with internal variables, and microscopic approaches based on kinetic theory or non-equilibrium ensemble formalisms [15].

Besides several advantages, the accuracy of the classical heat conduction model, known as Fourier’s law, is highly questioned, dealing with the micro-/nano-systems and biological tissues. In other words, the results obtained from the classical equations deviate from the available experimental data. It means that the continuum heat diffusion equation is insufficient and inappropriate for modeling heat transport in these cases. Consequently, the development of novel models to improve the results of the classical equation while being less computationally expensive and more simple to use is always a topic of interest. There are several techniques for modeling non-Fourier heat conduction. The dual-phase-lag (DPL) model as an accurate modified constitutive equation replacing the Fourier law to simulate the heat transport in special cases such as micro/nanoscales, ultra-fast laser-pulsed processes, living tissues, and carbon nanotube has been trendy [16].

### 1.3. Comparison between Fourier and Non-Fourier Heat Equations

The second law of thermodynamics is a valuable and universal tool to derive the generalizations of Fourier’s law. In many cases, only linear relations are considered between the thermodynamic fluxes and forces, i.e., the conduction coefficients are independent of the temperature. Kovacs and Rogolino [17] investigated a non-linearity in which the thermal conductivity depends on the temperature linearly. Additionally, that assumption was extended to the relaxation time, which appears in the hyperbolic generalization of Fourier’s law, namely, the Maxwell–Cattaneo–Vernotte (MCV) equation. Although such non-linearity in the Fourier heat equation is well-known, its extension onto the MCV equation is rarely applied. Since these non-linearities have significance from an experimental point of view, an efficient way is needed to solve partial differential equations. Table 1 summarizes a comparison between Fourier and Non-Fourier heat equations.

### 1.4. Results for Fourier and Non-Fourier Heat Equations via MATHEMATICA Software

In this section, the graphical results achieved via user-defined codes in “MATHEMATICA” software are provided. The sued-defined codes have been provided in Appendix A. The laser beam heats a sample in laser–target interaction, thus providing heat to the system (heat input). In the non-Fourier heat equation and Fourier equation, the heat transfer coefficient is the only parameter that provides heat output from the system, thus following the principle of the first law of thermodynamics. It is why the heat transfer coefficient has been considered in the non-Fourier heat equation, as mentioned in Appendix A. It is necessary to respect the law of conservation of energy. The parameters have been chosen based on the formalism provided for Fourier and non-Fourier heat equations with “SI” units. All the parameters are close to the experimental results [24].

**Figure 1:**
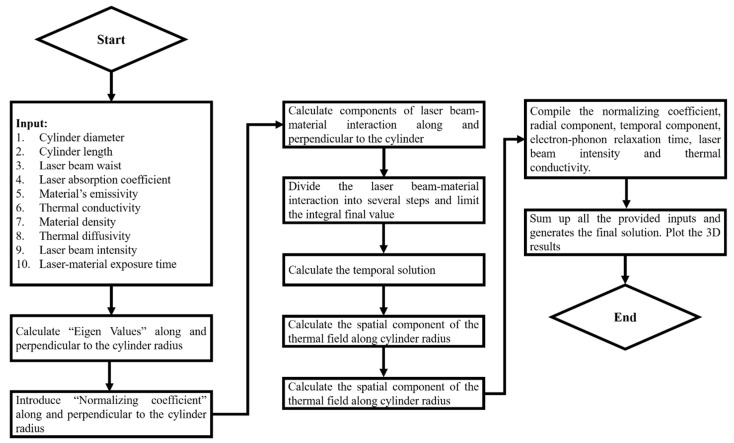
Flow chart to conduct simulation using Fourier heat equation.

Figure 1 shows the flow chart used to simulate in “MATHEMATICA” software for the Fourier heat equation. For each step, the codes are defined in Appendix A.1.

**Figure 2:**
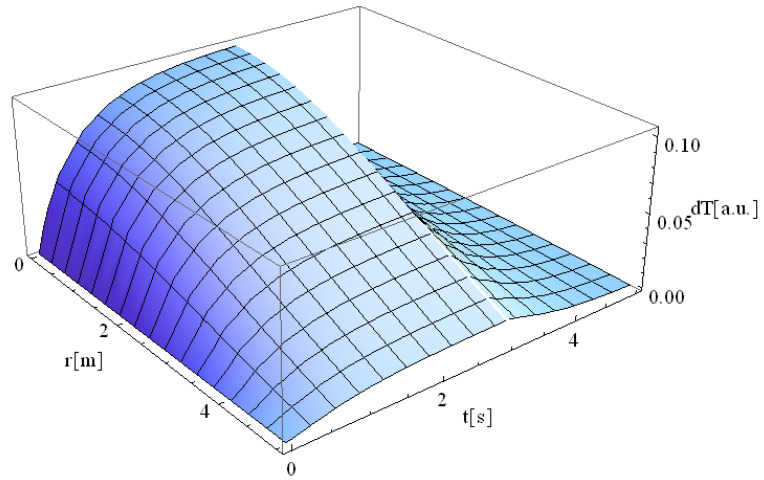
Results of the Fourier heat equation.

The above-defined flow chart was used to simulate the Fourier heat equation, and the result is shown in Figure 2. From this figure, it can be analyzed that the laser–material interaction lasts for 3 s, and after this time, the material starts cooling, which causes a sudden declination in temperature.

**Figure 3:**
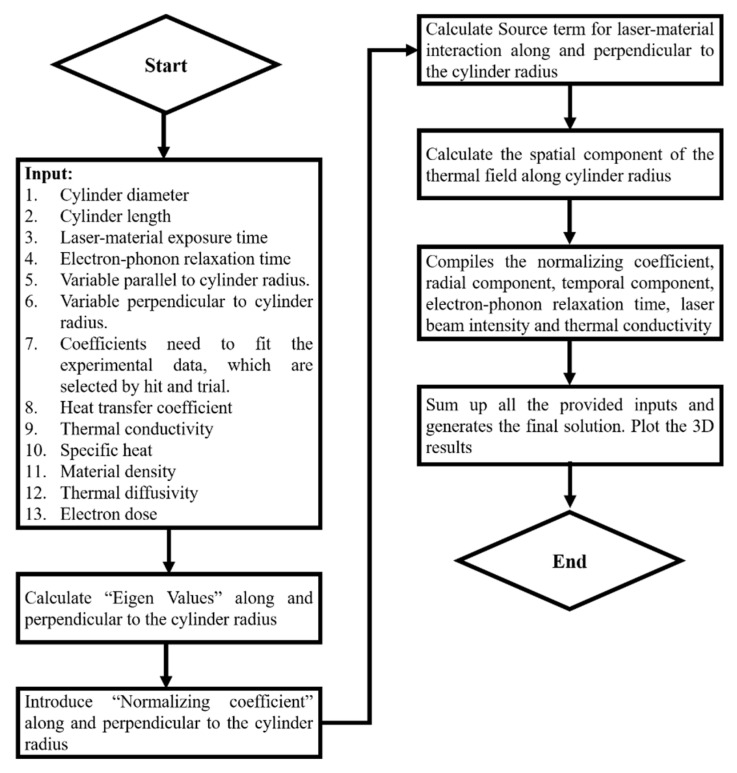
Flow chart to conduct simulation using non-Fourier heat equation.

Figure 3 shows the flow chart used to carry out the simulation in “MATHEMATICA” software for the non-Fourier heat equation. For each step, the codes are defined in Appendix A.2.

**Figure 4:**
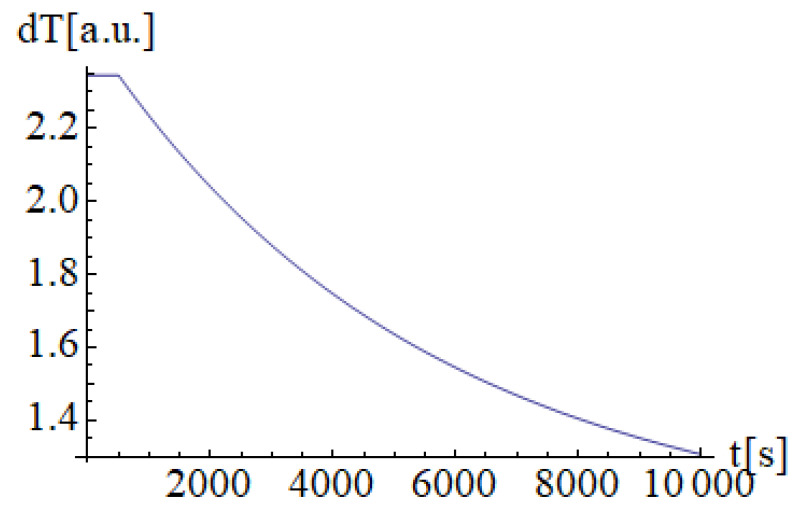
Results of the Non-Fourier heat equation.

Figure 4 shows the results after following the steps defined in Figure 3 when 500 s laser-material interaction time was selected. After material interaction, the thermal distribution intensity falls linearly, resulting in material cooling.

## 2. Various Applications of Integral Transform Technique (ITT): Fourier and Non-Fourier

In this section, efforts have been made to compile the processes in which ITT has been explored.

### 2.1. Metallic Materials

Metallic material is a category of materials that contain elemental metals and compounds and alloys [25,26]. There are 86 metals with different characteristic features among the 118 elements in the periodic table, and only a small fraction of these metals has engineering significance [27,28]. Over time, new processes for producing various materials with qualities superior to those of natural materials have been discovered [29,30]. Scientists are working to fully comprehend the links between the structural parts of materials and their qualities [31,32]. Changes in the relative proportions of the micro-constituents can produce drastic changes in terms of quality [33,34]. Phases are distinguished by their distinct crystal forms, compositions, and properties [35].

**Figure 5:**
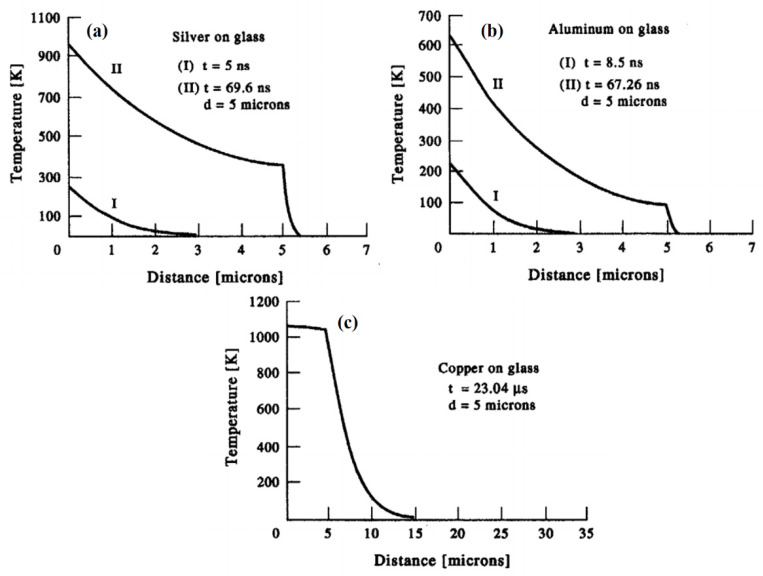
Temperature profile within a two-layer system with constant surface absorptance (**a**) silver thin film deposited on glass substrate at: (I) t = 5 ns; (II) t = 69.6 ns, (**b**) aluminum thin film deposited on glass substrate at two different exposure times: (I) t = 8.5 ns; (II) t = 67.26 ns, and (**c**) copper thin film deposited on glass substrate at exposure time t = 23.04 µs [38]; with permission from Elsevier.

Metallic materials have been used for various applications. Currently, laser cladding is usually applied in various industrial applications. For this purpose, Oane et al. [36] developed a model in which the phase transition from solid to liquid formation is considered with an absorption coefficient that can highlight the liquid generation during heating. A semi-analytical model was proposed, which considered the melt pool a sphere and solved the heat equation in spherical coordinates. They identified that an increase in the laser scanning speed does not affect the thermal distribution profile significantly. Besides, the simulation results were found in good agreement with the experimental analyses with a 7–15% mean deviation [37]. El-Adawi et al. [38] used ITT for laser–two layers interaction. The thermal distribution was estimated within the thin film and the substrate via mathematical formalism. Furthermore, the front surface temperature was also attained. The simulations were carried for the laser to (a) aluminum–glass, (b) silver–glass, (c) copper–glass, and (d) gold–glass interactions. It was found that the amount of absorbed power determined the crucial time necessary to begin melting. The studied profiles were no longer linear functions of the thermal characteristics of the two-layer system’s materials. The computed values for the crucial time determined whether or not a single laser pulse can cause the damage. The temperature profiles in the case of silver thin film deposited on glass substrate at: (I) t = 5 ns; (II) t = 69.6 ns, and the aluminum thin film deposited on glass substrate at two different exposure times: (I) t = 8.5 ns; (II) t = 67.26 ns, and copper thin film deposited on glass substrate at exposure time t = 23.04 µs are shown in Figure 5a,b.

A thermal interaction model for laser–metal was developed by Oane et al. [39]. The formalism’s primary purpose was to calculate the thermal distribution of electrons and phonons. In laser beam–metal interaction, the Fourier heat equation was solved to provide three-dimensional temperature fields, surface temperature, and steady-state quantum effects temperature. ITT was used to solve the Fourier heat equation. Experimental results for iron irradiation with an Nd: YAG laser beam (=355 nm wavelength) were used to validate the model. The model was able to estimate results with an accuracy of 20% deviation.

**Figure 6:**
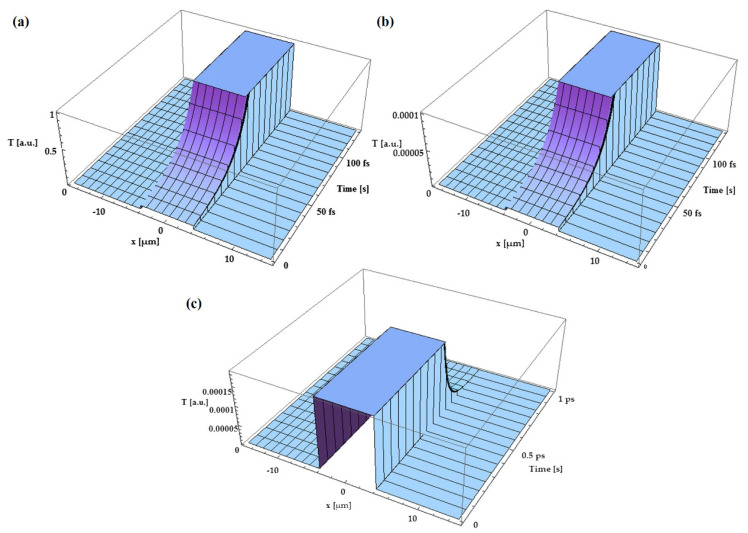
(**a**) Normalized electron thermal field in arbitrary units on the surface of an Au target versus time and distance. (**b**) Normalized electron thermal field in arbitrary units at distance = 4 µm, and (**c**) normalized electron thermal field in arbitrary units inside the Au target, at a depth of distance = 4 μm versus time (from 0 to 1 ps) and distance [40]; with permission from Elsevier.

Nicarel et al. [40] provided an analytical model for ultrafast thermal processes during femtosecond laser pulse–solid contact. An easy but powerful mathematical formalism was provided to analyze electron temperature’s spatial and temporal profiles under the irradiation with a single Gaussian femtoseconds laser pulse. A gold target was treated with 10^15^ W/cm^2^ laser pulses to demonstrate the model’s reliability. The simulations revealed that the electron temperature rises rapidly to saturation in the first 50 fs and that the heat does not travel far beyond the incident laser beam’s focal point. However, when moving from the top surface to the middle of the Au target, the thermal field diminishes by four orders of magnitude. After 1 ps of interaction time, the temperatures of electrons and phonons achieve equilibrium. After 200 fs, when the maximum penetration depth of 7 m is reached, the temperature rises to its highest point. Figure 6a–c show the normalized electron thermal field in arbitrary units on the surface of an Au target versus time and distance at a distance of 4 µm with respect to the sample surface, inside the Au target, and at a depth of 4 μm versus time (from 0 to 1 ps), respectively.

Serban et al. [41] employed a novel approach based on the Fourier heat equation to characterize the laser–metal thermal interaction. The 3D thermal field, surface temperature, and steady-state quantum effects of laser irradiation of metals were measured. An ITT was applied to the Anisimov and Nolte models for this purpose. The laser–gold thin film interaction was the subject of the simulations. The model was able to estimate outcomes with an accuracy of 10^−2^ K temperature after comparing with experimental data. In laser–metallic thin film interaction, Oane et al. [42] proposed an analytical technique for estimating thermal fields. A simplified model was presented for this purpose, which included the “global” heat equation. The major goal of this model was to take into account an absorption coefficient that was near to the real one. ITT was used to solve the heat equation for TEM_01_–silicon copper thin films. The findings revealed that (a) the thin-film absorption coefficient determines the thermal field, and (b) the length of the contact influences thermal distributions.

**Figure 7:**
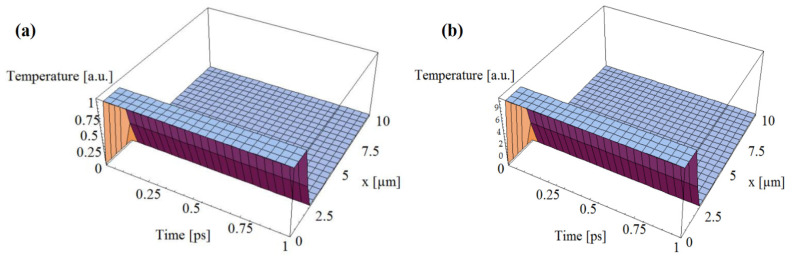
(**a**) Spatial–temporal distribution of the electrons’ thermal field generated by one laser pulse on an Au surface, when t = 100 fs and τ = 1 ps versus time (1 ps), and (**b**) influence of g/K value on temperature intensity at g = 1.05 × 10^16^ W/m^3^K, K = 315 W/mK, and laser pulse duration of 100 fs [19]; published under MDPI’s open access license.

Buca et al. [19] calculated the electron temperature fluctuation in metals due to the interaction of femtosecond laser pulses with metals. The classical Anisimov’s Two-Temperature Model was extended using the 3D telegraph Zhukovsky equation. The computational plots of electron thermal fields during the initial laser pulse interaction with a gold surface were deduced using this innovative approach. The interaction between the laser pulse and the metal sample during the initial picoseconds is governed by relaxation times and coupling factors over electron thermal conductivities. The higher the electron temperature, the lower the thermal conductivities. In contrast, the lower the electron temperature, the shorter the relaxation time. Figure 7a,b show the spatial–temporal distribution of the electrons’ thermal field generated by one laser pulse on an Au surface, when t = 100 fs and τ = 1 ps versus time (1 ps), and influence of g/K value on temperature intensity at g = 1.05 × 1016 W/m^3^K, K = 315 W/mK, and laser pulse duration of 100 fs.

Pelin et al. [43] implemented the ITT to solve Fourier and non-Fourier heat equations for the laser–nano-copper-particles interaction. The computations were carried out for 1, 2, and 4 particles-clusters. In comparison to the bulk material in pure form, placing groups or clusters of nano-particles-clusters on top of a layer exposed to irradiation results in a perceptible increase in temperature. Oane et al. [9] presented a new technique by combining the Two-Temperature Model with the Fourier heat equation to estimate the thermal distributions in electrons and phonons. The model was validated in the case of laser–gold, –copper, –silver, and –aluminum interactions. They determined that electron–phonon relaxation time plays a critical role in determining the precision of a given solution. In another study by Oane et al. [44], a semi-analytical model was presented for thermal fields induced in a small cylindrical sample made of tungsten. The model was validated with the experimental results for the tungsten irradiation by an electron beam with an energy of 6 MeV and average power of 62 W from a linear accelerator. The sample had diameter = 10 mm and length = 10 cm. The model predicted results with a mean absolute deviation of 10%.

### 2.2. Organic and Inorganic Materials

In modern chemistry, organic materials are described as carbon-based substances initially produced from living organisms but today include lab-synthesized counterparts [45]. The majority of them are made up of a few lightweight components, such as hydrogen, carbon, nitrogen, and oxygen [46]. Wood, feathers, leather, and synthetic materials are examples of organic materials [47]. Despite their diversity, they share some common qualities. For instance, prolonged exposure to light or other forms of radiation causes fading, yellowing, or embrittlement in many organic materials, which is caused by the breakdown of the covalent bonding structure shared by many carbon-containing molecules [48]. On the other hand, chemical substances that do not contain carbon are inorganic materials [49]. However, inorganic materials include elementary carbon and carbon compounds such as nitrogen, oxygen, and silicon [50].

Several pieces of research have been carried out on the ITT implementation in organic and inorganic materials. Using a hyperbolic heat conduction model, Vedavarz et al. [51] theoretically studied the transient temperature distributions in laser-irradiated materials. For temperature distributions, exact and limited mathematical solutions were generated, and significant factors are found. To account for the thermal wave’s finite speed, hyperbolic non-Fourier models were devised. The study considered laser surface interactions between two types of materials: (a) biological materials and (b) inorganic solids. They discovered that finite speed effects were substantial in short-pulse, where the laser input time is comparable to the material’s thermal characteristic time. Consequently, the resulting temperature variations were significantly different from those predicted by traditional infinite speed Fourier predictions. The ratio of thermal characteristic length to laser beam width was shown to be the most important parameter. Local temperature maxima or hot spots were detected at the beginning of the irradiation time with values > 0.2 in the range between 0.1 and 3, corresponding to different applications. The dual-phase lag (DPL) bio-heat transport equation was solved analytically by Kumar and Srivastava [52] using a finite-ITT (FITT). Three problems were formulated to show the applicability of the developed formalisms: (a) time-independent boundary conditions (persistent surface temperature), (b) time-dependent boundary conditions (sinusoidal surface heating), and (c) biological tissue irradiated with short-pulses. FITT-based analytical solutions of Fourier and non-Fourier heat conduction equations were linked with numerical solutions to calculate the thermal distribution. Thermal distribution intensity predicted by DPL was in between those achieved using the Fourier and hyperbolic heat conduction equations. When a comparison was made between DPL and Fourier heat models, the hyperbolic heat model generated more obvious wave characters in the anticipated temperature profiles. Talaee et al. [53] used separation of variables and Duhamel integral techniques to solve the three-dimensional hyperbolic heat conduction equation in a cubic media with rectangular cross-section under a pulsed heat flow on the top side. With both steady and pulsed fluxes, the closed-form solution of both Fourier and non-Fourier profiles was introduced. A model was created to simulate the interaction of a cubical tissue with a brief laser pulse. The Fourier and Non-Fourier temperature profiles showed a significant difference in the results. The findings can be used to treat biological tissues with lasers. To examine thermal damage in biological tissues, Zhou et al. [54] developed a thermal wave model of bioheat transmission and a seven-flux model for light propagation, and a rate process equation for tissue damage. They discovered that the thermal damage calculated using the thermal wave bioheat model differed significantly from that calculated using the traditional bioheat model. The assessment of thermal damage to biological tissue may not be reliable if the bioheat non-Fourier effect is not taken into account.

**Figure 8:**
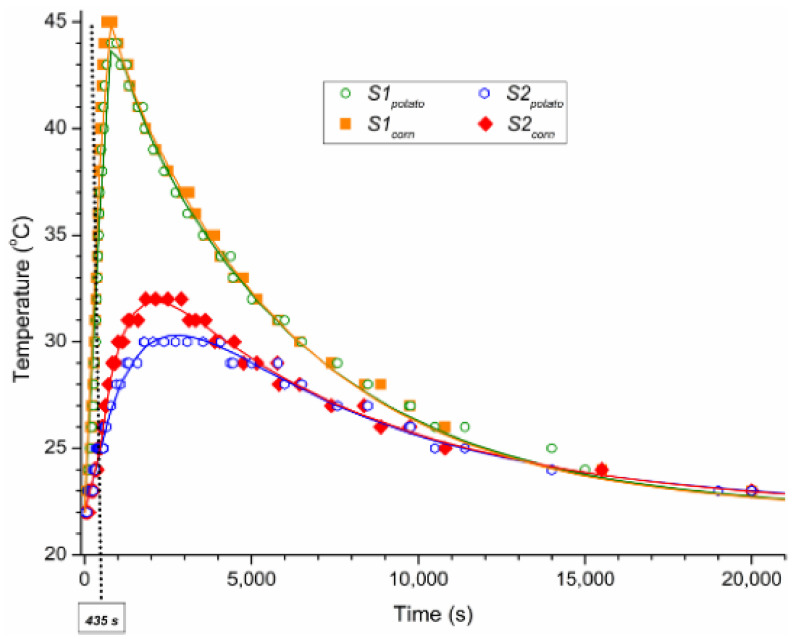
Temperature distribution in the irradiated potato and corn starches [55,56]; published under MDPI’s open access license.

Brasoveanu et al. [55,56] devised a mathematical model to represent the thermal distribution based on the Cattaneo–Vernote formalism’s heat equation, solved by ITT in finite domains. The model was used to calculate the relaxation time, the hottest point, and the peak thermal distribution intensity. Experiments were carried out to record the temperature distribution in granular starch exposed to ionizing radiation to validate the created model. The accelerated electrons irradiated corn starch with a mean energy of 5.5 MeV (=435 s). A temperature sensor was inserted within the starch sample to record the temperature during and after the irradiation. The relaxation time was discovered to play a crucial influence in the cooling of the irradiated sample. The hottest spot was found at a depth of 14 mm in the sample (100 mm total depth). Figure 8 shows the temperature distribution in corn and starch after irradiation.

### 2.3. Semiconducting Materials

A semiconductor is a type of crystalline substance that is in between a conductor and an insulator in terms of electrical conductivity [57]. Semiconductors are usually used to produce diodes, transistors, and integrated circuits, among other electronic devices [58]. Such devices have found widespread use because of their compactness, reliability, power efficiency, and low cost [59]. They have been used in power devices, optical sensors, and light emitters, including solid-state lasers, as discrete components [60]. They can handle a wide range of current and voltage, and, more importantly, they can be easily integrated into complicated yet easily manufactured microelectronic circuits [61]. They serve communications, signal processing, computing, and control applications in both consumer and industrial markets and will continue to be used in the foreseeable future [62].

**Figure 9:**
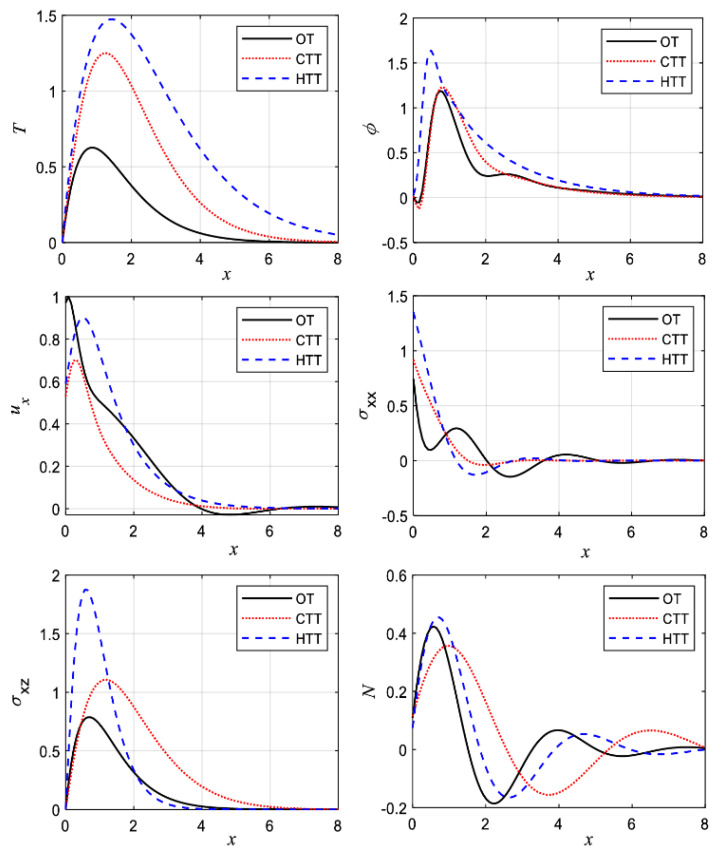
The main physical fields against the horizontal distance in generalized GL model with laser pulses and magnetic field in one temperature, two temperatures, and hyperbolic two temperatures [63]; with permission from Springer.

The ITT technique has also been explored in the semiconducting materials field. Mahdy et al. [63] identified the impact of three propagated waves: (a) elastic wave, (b) plasma wave, and (c) thermal waves using the hyperbolic generalized two-temperature theory. During the photothermal theory, the governing equations were investigated. They measured the effects of an external magnetic field and a laser pulse. The heat conductivity of semiconductor materials was examined. When the photothermal theory and the thermo-elasticity theory were connected, three different photo-thermo-elasticity models were developed. The principal equations were solved using the ITT in two-dimensional deformation. ITT was demonstrated using the double Fourier and Laplace transforms with appropriate conditions. The complete solutions were obtained numerically by inverting the double transforms with various thermal-elastic-mechanical-plasma boundary conditions. The photo-thermo-elasticity theory was used to compare three different models under an external magnetic field with variable thermal conductivity in the case of silicon material. Thermal memories with a negative thermal conductivity parameter constant, the effect of a magnetic field, and laser pulses in hyperbolic two temperatures were discovered to have a greater impact on the wave propagation of the primary fields. Figure 9 [63] shows the impact of three different cases according to the two-temperature parameter for the main physical fields against the horizontal distance. All numerical results in this category are made under the effect of the magnetic field and the laser pulses in the generalized Green and Lindsay (GL) model. The solid line curves in this category express the first case when the temperature (T) and angle (ϕ) are equal and when the heat supply is absent, which can be named the One-Temperature (OT) Model. The dotted lines curves refer to the Classical Two-Temperature Model (CTT), which is taken when the heat supply is also absent. The dashed lines curves show the general model, which is named the Hyperbolic Two-Temperature (HTT) Model. A clear significant effects in this figure are observed according to the three different cases of the hyperbolic two-temperature theory.

In another investigation by Mahdy et al. [64], the elastic semiconductor medium was irradiated with laser pulses. With the temporal fractional heat order, the laser pulses created vibrations in the medium’s inner structure. The effect of Hall current was observed when a strong external magnetic field was applied. In the case of silicon crystal material, the interaction between the strong magnetic field and the microstructure of the elastic medium was investigated. In the photothermal transport process, photo-excited electrons formed micro-temperature states. In the case of a semiconductor elastic rod, one-dimensional deformation was employed to describe the overlapping process between elastic-magneto-plasma-thermal distribution waves. In the field of microelements, the governing equations were solved utilizing the Laplace integral transform domain. Some external loads were applied to the medium’s outer free surface to achieve the physical quantities under examination. The experiment revealed that variations in laser pulse strength, fractional parameter, and Hall current significantly impacted physical quantities. When the propagated waves curves became similar with the increase in distance, all physical quantities reached equilibrium.

To simulate picosecond and femtosecond laser–silicon interactions, Xu and Wang [65] devised a lattice Boltzmann method (LBT). The LBT was used to calculate temperature fields compared to those derived using the parabolic heat conduction equation (PHCE) and the hyperbolic heat conduction equation (HHCE). Although the HHCE solved the PHCE’s infinite thermal propagation speed, it was unable to be applied to length scales comparable to the mean free path of energy carriers due to the failure of continuum techniques under severe non-equilibrium conditions. The LBT, which considers both impacts, might be used on both short and long-time scales. According to the LBT results, the speed of a thermal wave at the ballistic limit is equal to the speed of sound rather than the figure predicted by the HHCE, which is only true at the diffuse limit. It was further proved that utilizing the temperature gradient to calculate heat flow produces unreasonable results near the thermal wave front, whereas the LBM has no such drawback.

### 2.4. Graphene Material

A one-atom-thick layer of carbon atoms organized in a hexagonal lattice is known as “graphene” [66]. It is the building block of graphite, but graphene is a fascinating substance in and of itself, with a slew of astounding features that have earned it the moniker “wonder material” on numerous occasions [67]. With one-atom thickness, graphene is the thinnest material known to man and is roughly 200 times stronger than steel [68] at the same thickness scale. Furthermore, graphene is a good conductor of heat and electricity and has remarkable light absorption properties [69].

**Figure 10:**
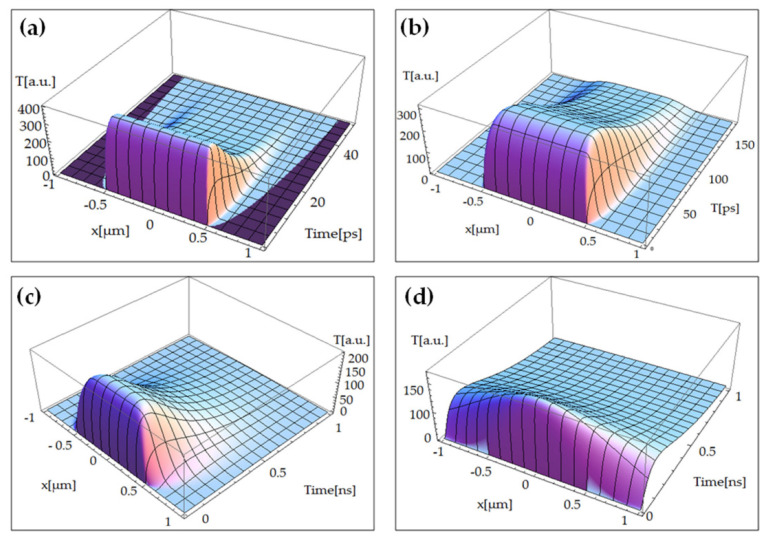
Temperatures field versus current coordinate and time during flash laser irradiation of a single-layer graphene of the (**a**) transverse optical phonons (TO), (**b**) longitudinal optical phonons (LO), (**c**) longitudinal acoustic phonons (LA), and (**d**) transverse acoustic phonons (TA) phonons [72]; published under MDPI’s open access license.

Scientists are working on ITT implementation in the case of graphene material. For this purpose, ITT was combined with the Anisimov–Nolte Two Temperature Model and the Cattaneo–Vernotte equation by Oane et al. [70]. For laser flash (TEM_00_)–graphene interaction, they used ITT to develop straightforward formulas for electron and phonon temperatures. Longitudinal optical phonons, transverse optical phonons, out-of-plane acoustic phonons, longitudinal acoustic phonons, transverse acoustic phonons, and out-of-plane optical phonons were all able to generate 3D thermal fields using the model. With a 15% variation, the simulated results were in good accord with the literature [71]. Buca et al. [72] proposed a Multiple-Temperature model to explain a single sheet of graphene irradiation with a flash laser. The Fourier heat equations based on quantum principles, including heat operators, were solved using Zhukovsky’s mathematical technique. Concerning traditional mathematics, simple solutions were inferred. Simple equations for electron and phonon temperatures were established in the case of flash laser treatment of a single layer of graphene. The findings were in good accord with those described in [73,74]. Figure 10a shows the phonon’s temperature, based upon the current model, for the transverse optical (TO) phonons branch. It can be observed that where the laser beam intensity is zero, the temperature distribution is zero. This shows the perfect coupling between Zhukovsky’s formalism and Multiple-Temperature Model (MTM). However, thermal effects can be clearly observed beyond the laser beam spot size (=1 µm), as a very short laser–graphene interaction time has been chosen (of ps order). The same phenomena can be analyzed in-case of Figure 10b,c related to longitudinal optical phonons (LO) and longitudinal acoustic phonons (LA) braches, respectively. As the simulation time has been increased, from ps to ns, the heat-wave finds enough time to transfer from the irradiated spot to the rest of the target. This result has been presented in Figure 10d in the case of transverse acoustic phonons (TA) branch.

However, the model presented a few drawbacks, such as:

- The duration of the irradiation time/pulse should be greater than 1 fs. When simulations are for attosecond pulse durations, the laser wavelength approaches the nucleus-first electron separation distance, resulting in meaningless results.
- The target size is limited to a range of 20–100 nm. The Fourier law collapses when the particle size is less than 20 nm, and reliable results are impossible to obtain.
- The model was restricted to single-layer graphene alone. The study of multilayer graphene becomes complicated to calculate since the absorption law and heat-transfer coefficients must be evaluated in real-time for each layer, necessitating the use of quantum-field theory in solid-state treatment.

Chan et al. [75] used the Fourier transformation approach to determine the orientation of hexagonal graphene domains on a Cu substrate. They devised a hexagon function to characterize hexagonal graphene’s diffraction pattern. In the frequency domain, hexagonal graphene domains produced on Cu (111) had an average orientation value of around 3°. The optical and electrical properties of a large-area graphene film (2 cm^2^) were tested for transparent conducting electrode applications. The findings showed that graphene produced on Cu (111) outperformed graphene developed on polycrystalline Cu.

## 3. Criticism on Dual-Phase-Lag (DPL) Heat Conduction and Guyer-Rumhansl Equations

The DPL and also the Guyer-Krumhansl equations are criticized from several different points of view. Negative temperatures Rukolaine [76,77] obtained an analytical solution for the DPL equation assuming a Gaussian initial condition. According to these calculations, the solutions present an unphysical behavior of temperature history, and it goes into the negative domain. Zhukovsky [78,79] achieved a similar conclusion for the GK equation. Wang et al. [80] tested the Thermo mass and DPL among other different heat conduction models by calculating Taitel’s problem [81]. Inconsistent, unphysical behavior is shown, and the temperature achieves the negative domain again. It is an old problem, also mentioned several times for the Maxwell-Cattaneo-Vernotte equation.

*Time shift paradox*: the delayed form of the DPL model directly contradicts the requirement of the homogeneity of time. One can transform the equation to a single-phase-lag equation by shifting the zero point of *t*, the starting point of the time measurement. The proper mathematical representation of non-relativistic time considering all expected properties is a one-dimensional affine space, and with that model cannot have two relaxation times; only their difference plays a role [82].

*Mathematical question*: in the literature, the DPL-type constitutive equations are analyzed to prove the uniqueness and well-posedness of a process driven by such a constitutive equation. It is found by Fabrizio et al. [83,84,85] that there are mathematical conditions beyond the physical ones to obtain an exponentially stable equilibrium solution for the DPL equation. Such a condition requires a negative time delay (called a retarded effect) between the heat flux and temperature gradient. It is important because Tzou in Ref. [86] directly interprets both cases with the cause–effect concept, i.e., the quantity with the higher relaxation time is the effect caused by the other one. As Fabrizio states [83], the DPL model can be rewritten with the time delay difference, leading to a single-phase-lag model, but the temperature gradient has a relaxation time < 0. The opposite case is mathematically ill-posed, which enlightens the validity of the Maxwell–Cattaneo–Vernotte (MCV) equation but excludes equations based on arbitrary Taylor series expansion. Quintanilla et al. [87,88] obtain the same conclusion regarding the relaxation times and the ill-posedness. However, exponential stability seems to be a too-strict requirement, and the asymptotic stability of homogeneous equilibrium is satisfied only with non-negative coefficients. The condition of asymptotic stability is also reasonable from a physical point of view [89,90,91].

*Second law*: Fabrizio and co-workers also checked thermodynamical restriction. It was tested by using Clausius–Duhem inequality on cyclic histories. The conditions of exponential stability for the DPL equation turned out to be too strict [84]. On the other hand, the experimental pieces of evidence mostly show that this difference is negative. In the work of Liu and Chen [92], the DPL equation is fitted to experimental results, and in heterogeneous materials, there is a similar situation [93,94]. It draws attention to the practical aspects, which are emphasized further in the next section.

## 4. Future Outlook

Fourier and Non-Fourier heat equations’ solutions using the integral transform technique (ITT) have proved their viability in the interactions of laser–various materials. In the authors’ opinion, there are various domains where these equations can be still explored, as:

- Fourier and non-Fourier heat equations can be linked with Two- and Multi-Temperature Models to estimate the thermal distributions within the sample. The Two-Temperature Model can yield thermal distribution in electrons and phonons, while the Multi-Temperature Model can give thermal distributions in longitudinal optical phonons, transverse optical phonons, out-of-plane acoustic phonons, longitudinal acoustic phonons, transverse acoustic phonons, and out-of-plane optical phonons.
- These heat equations have not been explored in the case of laser–ceramic interaction. Ceramics are porous materials having voids and pores of various dimensions [95]. Furthermore, ceramics behave as insulators and present complex rheological properties [96]. Hence, there is a need to modify the Fourier and non-Fourier heat equations by taking into considerations all the aspects explained above. It will open a new area for laser–ceramic interaction heat equations’ solution using ITT. Furthermore, it is a long-lasting task to understand heat conduction phenomena beyond Fourier. Besides the low-temperature experiments on extremely pure crystals, it has been turned out recently that heterogeneous materials with macro-scale size can also show thermal effects that the Fourier equation cannot model. It is known as over-diffusive propagation, different from low-temperature observations, and is found in numerous samples made from metal foam, rocks, and composites. The measured temperature history is similar to what Fourier’s law predicts, but the usual evaluation cannot provide reliable thermal parameters. Feher et al. [97] reported experiments on several rock types, each having multiple samples with different thicknesses. They showed that the size-dependent thermal behavior could occur for both Fourier and non-Fourier situations. Moreover, based on the experimental data, they found an empirical relation between the Fourier and non-Fourier parameters, which could be helpful in later experiments to develop a more robust and reliable evaluation procedure.
- Another domain is to develop a solution for laser–metal matrix composites (MMCs) interaction [98]. MMCs are the composition of metals and ceramics mixed to achieve a combination of metallic and ceramic properties [99]. In this area, no Fourier and non-Fourier models are dealing with laser–MMCs interaction [100]. From the authors’ point of view, this problem can be solved by separately solving the heat equations for metals and ceramics. Following, “superposition” must be implemented based on the percentage of metals and ceramics within MMCs to attain the final solution.
- For the automation of Fourier and non-Fourier heat equations, these equations can be linked with artificial neural networking (ANN). A series of computations can be carried out based on inputs and corresponding outputs [101]. These values can be used to train ANN and further used to estimate output based on desired inputs [102]. Recently, Mahmood et al. used the same approach to link ANN with analytical modeling for process automation [103].
- This method has been explored for laser–metal, Two-Temperature Model for laser–metal interaction, ultra-short laser–material interaction, and electron beam–organic and –inorganic interaction. However, there are various applications that have not been studied in detail. For instance, the laser–graphene interaction, laser–ceramic reinforced metal matrix interaction, laser–nanoparticles interaction and laser–ceramic interaction are a few subjects of interest in the near future.

The authors believe that the Fourier and non-Fearer heat equations will exponentially gain attention from scientists and researchers after exploring the areas mentioned above.

## 5. Conclusions

Fourier and non-Fourier heat equations can estimate the thermal distribution and phase transformation based on the provided operating conditions. For this purpose, this review article discusses the heat equations, including Fourier and non-Fourier heat equations. An evaluation is presented between Fourier and non-Fourier heat equations. Besides, their generalized solutions have been presented. Various components of heat equations and their implementation in the various processes have been illustrated. For solving these equations, “MATHEMATICA” software codes have been provided for the scientific community. Furthermore, various applications of Fourier and non-Fourier techniques have been provided. Based on the current study, the following conclusions have been deduced:

- The Fourier heat equation is simple to use but involves infinite heat propagation compared to the non-Fourier heat equation. It can be applied to both finite and infinite mediums; however, it yields excellent results with a finite medium. It does not involve electron–phonon relaxation time. The Fourier heat equation cannot be linked with the Two-Temperature Model in a natural way.
- The non-Fourier heat equation is complex and involves various unknowns in comparison to the Fourier heat equation. It can deal with both finite and infinite mediums, but in the current scenario, the non-Fourier heat equation works well in the finite medium. However, it depends on the user’s choice to implement the non-Fourier heat equation in finite and infinite mediums. On the other hand, one should recognize that ITT works well only on the finite target. It takes into account the electron–phonon relaxation time. To achieve an optimum solution, experimental data are always needed for normalizing coefficients. The non-Fourier heat equation can be linked with the Two-Temperature Model in a more efficient way.
- Fourier and non-Fourier heat equations can follow equilibrium and non-equilibrium thermodynamics models for ultra-short laser heating. The classical heat conduction model’s accuracy is highly questioned, dealing with the micro-/nano-systems and biological tissues. In simple words, the results obtained from the classical equations deviate from the available experimental data. It means that the continuum heat diffusion equation is insufficient and inappropriate for modeling heat transport in these cases.
- Fourier and Non-Fourier heat equations have proved their reliability in the case of laser–metallic materials, electron beam–biological and –inorganic materials, laser–semiconducting materials, and laser–graphene material interactions. The presented simulation results showed a strong correspondence with experimental results. It is worth mentioning that the accuracy and transient behavior determine the accuracy of Fourier and non-Fourier heat equations. Especially in the case of biological systems, the Fourier model is not necessarily far from the measurements, but the transient behavior could be significantly different, and therefore the prediction is questionable.
- Through the literature survey and the authors’ research experience, it has been identified that the material properties play an essential role in defining the precise results of Fourier and non-Fourier heat equations.
- In the case of laser–graphene interaction, there is a certain restriction of the integral transform technique. The duration of the irradiation time/pulse should be greater than 1 fs. When computations are for attosecond pulse durations, the laser wavelength approaches the nucleus-first electron separation distance, resulting in meaningless results. The study of multilayer graphene becomes complicated to calculate since the absorption law and heat-transfer coefficients must be evaluated in real-time for each layer, necessitating the use of quantum-field theory in solid-state treatment.

## Figures and Tables

**Table 1:** Comparison between Fourier and non-Fourier heat equations.

Fourier Heat Equation	References	Non-Fourier Heat Equation	References
This equation is simple to use but involves infinite heat propagation in comparison to the non-Fourier heat equation.	[18]	This equation is complex and involves various unknowns in comparison to the Fourier heat equation.	[19]
It can be applied to both finite and infinite mediums; however, it yields excellent results with a finite medium.	[20]	It can deal with both finite and infinite mediums, but in the current scenario, the non-Fourier heat equation works well in the finite medium. However, it depends on the user’s choice to implement the non-Fourier heat equation in finite and infinite mediums. On the other hand, one should recognize that ITT works well on the finite target only.	[21]
It does not involve electron–phonon relaxation time.	[22]	It takes into account the electron–phonon relaxation time.	[22]
		To achieve an optimum solution, experimental data are always needed for normalizing coefficients.	[23]
The Fourier heat equation cannot be naturally linked with the Two-Temperature Model.	[8,9]	The non-Fourier heat equation can be linked with the Two-Temperature Model more efficiently.	[8,9]
Fourier and non-Fourier heat equations can follow equilibrium and non-equilibrium thermodynamics models for ultra-short laser heating.			
The classical heat conduction model’s accuracy is highly questioned, dealing with the micro-/nano-systems and biological tissues. In simple words, the results obtained from the classical equations deviate from the available experimental data. It means that the continuum heat diffusion equation is insufficient and inappropriate for modeling heat transport in these cases.			

**Figure A1:**
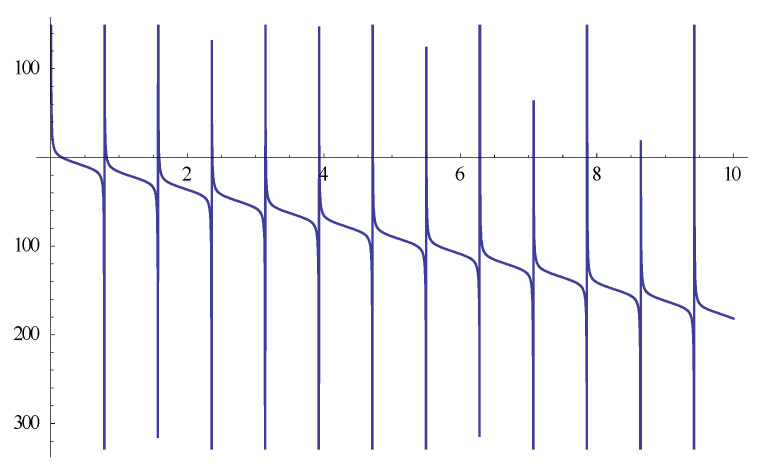
Plot for Eigen Values along the cylinder radius using Equation (A1).

**Figure A2:**
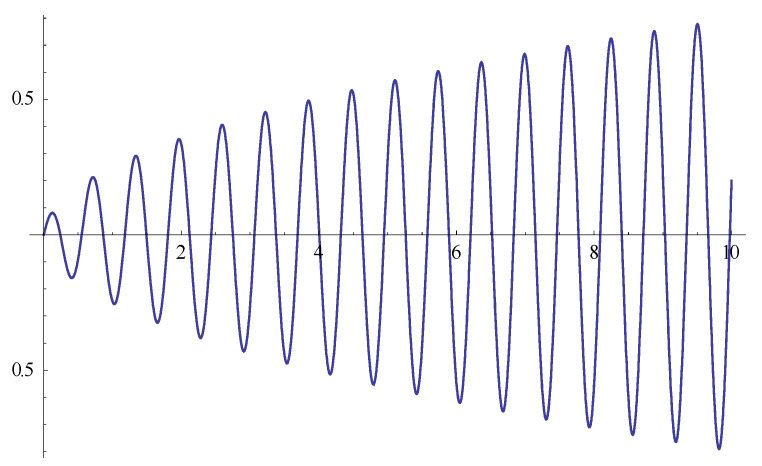
Plot for Eigen Values perpendicular to the cylinder radius using Equation (A2).

## Data Availability

Not applicable.

## Appendix A

### Appendix A.1. Fourier Heat Equation

For the Fourier illustration, laser–solid cylinder interaction has been considered. The user-defined codes utilized in “MATHEMATICA” software have been compiled in Table A1.

**Table A1:** Fourier heat equation with “MATHEMATICA” software user-defined codes with an illustration.

Sr. No.	MATHEMATICA Syntax	Explanation
01.	**a** = 4;	Cylinder diameter.
02.	**b** = 10;	Cylinder length.
03.	**w** = 4;	Laser beam waist.
04.	**α** = 0.00005;	Laser beam linear absorption coefficient.
05.	**h** = 0.02;	Material’s emissivity.
06.	**c** = 0.356;	Material’s specific heat.
07.	**k** = 0.018;	Thermal conductivity.
08.	$\rho =5.27*\left(10^{-3}\right);$	Material’s density.
09.	$\gamma =\frac{k}{c}*\rho ;$	Thermal diffusivity.
10.	**in** = 10;	Laser beam intensity.
11.	**t0** = 3;	Laser–material exposure time.
12.	**vbessel** = {0.0018,0.4,0.7,1,1.3,1.6,1.9,2.2,2.5,2.8};	Eigen Values along the cylinder radius.
13.	**vcos** = {0.0029,0.8,1.6,2.4,3.2,4,4.8,5.6,6.2,7};	Eigen Values perpendicular to the cylinder radius.
14.	**wc1** = **Table**[**1**/**4**(**Part**[**vcos**,***j***])^**3**(**2*h***(**Part**[**vcos**,***j***]) + **2*ah***^**2**(**Part**[**vcos**,***j***]) + **2*a***(**Part**[**vcos**,***j***])^**3** − **2*h***(**Part**[**vcos**,***j***])**Cos**[**2*a***(**Part**[**vcos**,***j***])] − ***h***^**2Sin**[**2*a***(**Part**[**vcos**,***j***])] + (**Part**[**vcos**,***j***])^**2Sin**[**2*a***(**Part**[**vcos**,***j***])]),{***j***,**10**}]; **mih** = Table[BesselJ[0,(Part[vbessel,i]) * 10]^2,{i,10}]; **wm** = Table[N[Part[mih,i]],{i,10}]; **poc** = Table[((2 * (Part[vbessel,i])^2)^−1) * ((b * h)^2 + (b * Part[vbessel,i])^2),{i,10}]; **wc2** = Table[Part[poc,i] * Part[wm,i],{i,10}];	“wc1 to wc2” are the normalizing coefficients perpendicular and along the cylinder radius.
15.	**int** = Table[BesselJ[0,(Part[vbessel,i]) * y] * y * Exp[−y^2/(w^2)],{i,10}]; **op** = Table[FunctionInterpolation[Part[int,i], {y, 0, 10}],{i,10}]; **ps** = Table[Integrate [Part[op,i][y], {y, 0, 10}],{i,10}];	Components of laser beam–material interaction along the cylinder radius.
16.	$\begin{array}{l} noi=Table[(Cos\left[Part\left[vcos,j\right]*x\right]\\ \;\;\;+\left(h/\left(Part\left[vcos,j\right]\right)\right)\\ \;\;\;*Sin\left[Part\left[vcos,j\right]*x\right])\\ \;\;\;*Exp\left[-\alpha *x\right],\left\{j,10\right\}]; \end{array}$	Components of laser beam–material interaction perpendicular to the cylinder radius.
17.	**zz1** = Chop[Integrate[Part[noi,1],{x,0,4}]]; **zz2** = Chop[Integrate[Part[noi,2],{x,0,4}]]; **zz3** = Chop[Integrate[Part[noi,3],{x,0,4}]]; **zz4** = Chop[Integrate[Part[noi,4],{x,0,4}]]; **zz5** = Chop[Integrate[Part[noi,5],{x,0,4}]]; **zz6** = Chop[Integrate[Part[noi,6],{x,0,4}]]; **zz7** = Chop[Integrate[Part[noi,7],{x,0,4}]]; **zz8** = Chop[Integrate[Part[noi,8],{x,0,4}]]; **zz9** = Chop[Integrate[Part[noi,9],{x,0,4}]]; **zz10** = Chop[Integrate[Part[noi,10],{x,0,4}]]; zz={zz1,zz2,zz3,zz4,zz5,zz6,zz7,zz8,zz9,zz10};	Divide the laser beam–material interaction into several steps and limits the integral’s final value. Without “chop”, the integral will result in a very high value.
18.	**b****4** = Table[g * (Part[vcos,j]^2+Part[vbessel,i]^2),{i,10},{j,10}]; **ft10** = Table[(1 - Exp[ −(Part[b4,i,j]) * t]) − (1 − Exp[ −(Part[b4,i,j]) * (t − t0) * UnitStep[t − t0]]),{i,10},{j,10}]; **pr4** = Table[(Part[vcos,j]^2+Part[vbessel,i]^2)^ −1,{i,10},{j,10}]; **timpul** = Table[Part[pr4,i,j] * Part[ft10,i,j],{i,10},{j,10}];	Calculates the temporal solution; t is the computational time.
19.	radial=Table[BesselJ[0,(Part[vbessel,i])∗r],{i,10}];	The spatial component of the thermal field along cylinder radius.
20.	$hai=Table\left[\left(Part{\left[wc1,j\right]}^{-1}\right)*\left(Part{\left[wc2,i\right]}^{-1}\right)\;\;\;\;\;\;\;\;\;\;*Part\left[radial,i\right]*Part\left[timpul,i,j\right]\;\;\;\;\;\;\;\;\;*Part\left[zz,j\right]*Part\left[ps,i\right]*\alpha \;\;\;\;\;\;\;\;\;\;\;*in/k,\left\{i,10\right\},\left\{j,10\right\}\right];$	Compiles the normalizing coefficient, radial component, temporal component, Lambert–Beer law, material absorption coefficient, laser beam intensity, and thermal conductivity.
21.	snoopy=Sum[Part[hai,i,j],{i,10},{j,10}];	Sums up every provided input and generates the final solution; *i* and *j* are the number of steps used in simulations.
22.	**j** = Plot3D[%,{r,0,5},{t,0,5},AxesLabel->{“r[m]”,”t[s]”,”dT[a.u.]”},AxesStyle->Directive[20]] Show[j,Shading->False]	Plot and gives 3D results.

The Eigen Values named “vbessel and vcos” were calculated using Equations (A1) and (A2), respectively, as [104].

(A1) $$2\cot\left(\lambda_{j}a\right)=\frac{\lambda_{j}k}{h}-\frac{h}{\lambda_{j}k}.$$

(A2) $$\mu_{i}j_{o}^{’}\left(\mu_{i}b\right)+hj_{o}\left(\mu_{i}b\right)=0.$$

Here, *k* is the thermal conductivity, *h* is the heat transfer coefficient, $\lambda_{j}$ is the Eigenvalue, $j_{o}^{’}$ is the Bessel function of zero-order derivative and $j_{o}$ is the Bessel function of zero-order. These equations were written in “MATHEMATICA” software and a technique was applied to calculate Eigen Values, as shown in Table A2.

**Table A2:** User-defined code in “MATHEMATICA” software with illustration utilized to calculate the Eigen Values based on Equations (A1) and (A2).

Step No.	MATHEMATICA Syntax	Explanation
01.	Plot[2∗Cot[j∗a]−j∗k/h+h/(j∗k),{j,0,10}]	It will give a plot between the Eigen Function (Equation (A1)) and the first 10 Eigen Values along the cylinder radius.
02.	Figure A1	The plot is achieved from step 1. Using Figure A1, one can observe that the function value is repeating itself after a certain interval along the *j*-axis. The Eigen Values have been calculated against these *j*-values.
03.	FindRoot[2∗Cot[j∗4]−j∗k/h+h/(j∗k)==0,{j,1}]	Each value of the *j*-axis has to be inserted manually in the defined syntax “{*j*,1}” to calculate the corresponding Eigen Value. Here, “1” is the first Eigen Value along the *x*-axis utilized from Figure A1.
04.	Plot[x∗BesselJ[0,b∗x]+h∗BesselJ[0,b∗x],{x,0,10}]	It will result in a plot between the Eigen Function (Equation (A2)) and the first 10 Eigen Values perpendicular to the cylinder radius.
05.	Figure A2	It is the result of step 4. From Figure A2, one can observe that the function value is repeating itself after a certain interval along the *x*-axis. The Eigen Values have been calculated against these *x*-values.
06.	**FindRoot**[−x * BesselJ[0,10 * x]+h * BesselJ[0,10 * x]==0,{x,0.5}]	Each value of the *x*-axis has to be inserted manually in the defined syntax “{*x*,0.5}” to calculate the corresponding Eigen Value. Here, “0.5” is the first Eigen Value along the *x*-axis utilized from Figure A2.
The authors identified that 10 Eigen Values along the radius and 10 Eigen Values perpendicular to the radius of the cylinder, resulting in a total of 100 values, are enough to achieve an answer with a precision of 0.01 K.		

### Appendix A.2. Non-Fourier Heat Equation

For the non-Fourier case, electron beam–starch cylinder interaction has been discussed. The user-defined codes with illustrations for “MATHEMATICA” software have been collected in Table A3.

**Table A3:** Non-Fourier heat equation “MATHEMATICA” software user-defined codes with an explanation.

Sr. No.	MATHEMATICA Syntax	Explanation
01.	**a** = 0.1;	Cylinder diameter.
02.	**b** = 0.06;	Cylinder length.
03.	**t0** = 500;	Laser–material exposure time.
04.	**tau** = 5000;	Electron–phonon relaxation time.
05.	**Rv** = 0;	Variable parallel to cylinder radius.
06.	**Zv** = 0.055;	Variable perpendicular to cylinder radius.
07.	**c1** = **c2** = 1;	Coefficients needed to fit the experimental data, which are selected by hit and trial.
08.	**h** = 0.3;	Heat transfer coefficient.
09.	**K** =0.078;	Thermal conductivity.
10.	**c** = 977;	Specific heat.
11.	ro=541;	Material density.
12.	gamma=k/(c∗ro);	Thermal diffusivity.
13.	**w** = 23.8 * 10^−3; **unu** = 0.96; **zo** = 43.2 * 10^−3; **q** = 4.7 * 10^−3; **debit** = 200; **fr** = Exp[−r * r/(2 * w * w)]; **fz** = unu/(1+Exp[z/q − zo/q]); **s** = debit * ro * fz * fr;	Here, “w” is the laser beam waist, “z_o_” is the maximum distance that electrons can penetrate, “q” is the electron beam waist perpendicular to the cylinder and “debit” is the electron dose on a given material.
14.	**vbessel** = {2.3556, 5.4711, 8.6048, 11.742, 14.882, 18.022, 21.162, 24.303, 27.444, 30.585 };	Eigen Values along the cylinder radius.
15.	**vcos** = {4.898087,8.65222, 12.4238, 16.2351, 20.0775, 23.9414,27.8201, 31.7093, 47.324, 51.2361 };	Eigen Values perpendicular to the cylinder radius.
16.	**mih** = Table[BesselJ[0,(Part[vbessel,i]) * b]^2,{i,10}]; wm = Table[N[Part[mih,i]],{i,10}]; **poc** = Table[((2 * (Part[vbessel,i])^2)^-1) * ((b * h/k)^2+(b * Part[vbessel,i])^2),{i,10}]; **cnr1** = Table[Part[poc,i] * Part[wm,i],{i,10}]; **cnz2** = Table[1/(4(Part[vcos,j])^3)((2(h/k) (Part[vcos,j])+2 a (h/k)^2(Part[vcos,j])+2 a(Part[vcos,j])^3-2(h/k)(Part[vcos,j])Cos[2a(Part[vcos,j])]-(h/k)^2Sin[2a(Part[vcos,j])]+ (Part[vcos,j])^2Sin[2 a (Part[vcos,j])])),{j,10}];	“cnr1 to cnr2” are the normalizing coefficients perpendicular and along the cylinder radius.
17.	**pz** = Table[fz * Part[kz,j],{j,10}]; **pr** = Table[fr * r * Part[kr,i],{i,10}]; **zz1** = Chop[Integrate[Part[pz,1],{z,0,a}]]; **zz2** = Chop[Integrate[Part[pz,2],{z,0,a}]]; **zz3** = Chop[Integrate[Part[pz,3],{z,0,a}]]; **zz4** = Chop[Integrate[Part[pz,4],{z,0,a}]]; **zz5** = Chop[Integrate[Part[pz,5],{z,0,a}]]; **zz6** = Chop[Integrate[Part[pz,6],{z,0,a}]]; **zz7** = Chop[Integrate[Part[pz,7],{z,0,a}]]; **zz8** = Chop[Integrate[Part[pz,8],{z,0,a}]]; **zz9** = Chop[Integrate[Part[pz,9],{z,0,a}]]; **zz10** = Chop[Integrate[Part[pz,10],{z,0,a}]]; **zz** = {zz1, zz2, zz3, zz4, zz5, zz6, zz7, zz8, zz9, zz10};	Source term for laser–material interaction perpendicular to the cylinder radius.
18.	**rr1** = N[Integrate[Part[pr,1],{r,0,b}]; **rr2** = N[Integrate[Part[pr,2],{r,0,b}]]; **rr3** = N[Integrate[Part[pr,3],{r,0,b}]]; **rr4** = N[Integrate[Part[pr,4],{r,0,b}]]; **rr5** = N[Integrate[Part[pr,5],{r,0,b}]]; **rr6** = N[Integrate[Part[pr,6],{r,0,b}]]; **rr7** = N[Integrate[Part[pr,7],{r,0,b}]]; **rr8** = N[Integrate[Part[pr,8],{r,0,b}]]; **rr9** = N[Integrate[Part[pr,9],{r,0,b}]]; **rr10** = N[Integrate[Part[pr,10],{r,0,b}]]; **rr** = {rr1,rr2,rr3,rr4,rr5,rr6,rr7,rr8,rr9,rr10}; $psij=\mathrm{Table}\left[\left(\mathrm{debit}*\mathrm{ro}*\mathrm{unu}/\left(\mathrm{Part}\left[\mathrm{cnr}1,i\right]*\mathrm{Part}\left[\mathrm{cnz}2,j\right]\right)\right)\;*\mathrm{Part}\left[\mathrm{rr},i\right]\;*\mathrm{Part}\left[\mathrm{zz},j\right]/\left(\mathrm{Part}{\left[\mathrm{vbessel},i\right]}^{2}+\mathrm{Part}{\left[\mathrm{vcos},j\right]}^{2}\right),\left\{i,10\right\},\left\{j,10\right\}\right];$	Source term for laser–material interaction along the cylinder radius.
19.	**radical** = Table[Sqrt[1/gamma^2 − (4 * tau/gamma) * Part[vbessel,i]^2 − (4 * tau/gamma) * Part[vcos,j]^2],{i,10},{j,10}];	Spatial component of the thermal field along cylinder radius.
20.	**krv** = Table[BesselJ[0,(Part[vbessel,i]) * rv],{i,10}]; **kzv** = Table[(Cos[Part[vcos,j] * zv] + (e/(k * Part[vcos,j])) * Sin[Part[vcos,j] * zv]),{j,10}]; $\begin{array}{ll} Tij=\mathrm{Table}[(c1 & *\mathrm{Exp}[\left(-\left(1/\mathrm{gamma}\right)-\mathrm{Part}\left[\mathrm{radical},i,j\right]\right)\\ & *\left(t-t0\right)*\mathrm{UnitStep}\left[t-t0\right]\\ & *\mathrm{gamma}/\left(2*\mathrm{tau}\right)]+c2\\ & *\mathrm{Exp}[\left(-\left(1/\mathrm{gamma}\right)+\mathrm{Part}\left[\mathrm{radical},i,j\right]\right)*t\\ & *\mathrm{gamma}/\left(2*\mathrm{tau}\right)])\\ & *\mathrm{Part}\left[\mathrm{krv},i\right]*\mathrm{Part}\left[\mathrm{kzv},j\right],\left\{i,10\right\},\left\{j,10\right\}]; \end{array}$ **Tij1** = Table[c1 * Exp[(−(1/gamma) − Part[radical,i,j]) * (t − t0) * UnitStep[t − t0] * gamma/(2 * tau)] * Part[krv,i] * Part[kzv,j],{i,10},{j,10}]; **Tij2** = Table[c2 * Exp[(−(1/gamma) + Part[radical,i,j]) * (t−t0) * UnitStep[t−t0] * gamma/(2 * tau)] * Part[krv,i] * Part[kzv,j],{i,10},{j,10}]; **Tij** = Tij1 + Tij2; **Tsj0** = Table[ Part[psij,i,j] * Part[krv,i] * Part[kzv,j],{i,10},{j,10}]; **Tsij1** = Table[ Exp[(−(1/gamma)−Part[radical,i,j]) * (t − t0) * UnitStep[t − t0] * gamma/(2 * tau)] * Part[krv,i] * Part[kzv,j],{i,10},{j,10}]; **Tsij2** = Table[ Exp[(−(1/gamma) + Part[radical,i,j]) * (t−t0) * UnitStep[t − t0] * gamma/(2 * tau)] * Part[krv,i] * Part[kzv,j],{i,10},{j,10}]; **Tsij** = Tsj0 + Tsij1 + Tsij2; **T** = Sum[Part[Tij,i,j],{i,1},{j,1}];	Compiles the normalizing coefficient, radial component, temporal component, electron–phonon relaxation time, laser beam intensity, and thermal conductivity.
21.	**Tsursa** = Sum[Part[Tsij,i,j],{i,10},{j,10}]; **Plot**[%%,{t,0,10000}, AxesLabel->{“t[s]”,”dT[a.u.]”},AxesStyle->Directive[20]] **Plot**[%%,{t,0,10000},AxesLabel->{“t[s]”,”dT[a.u.]”},AxesStyle->Directive[20]]	Sums up every provided input and generates the final solution, *i* and *j* are the number of steps used in simulations. Plots and gives 3D results.
The Eigen Values “vbessel” and “vcos” were calculated using the same procedure provided in Table A2.		
